# Hybrid Ablation of Atrial Fibrillation: A Contemporary Overview

**DOI:** 10.3390/jcdd9090302

**Published:** 2022-09-08

**Authors:** Massimiliano Marini, Luigi Pannone, Domenico G. Della Rocca, Stefano Branzoli, Antonio Bisignani, Sahar Mouram, Alvise Del Monte, Cinzia Monaco, Anaïs Gauthey, Ivan Eltsov, Ingrid Overeinder, Gezim Bala, Alexandre Almorad, Erwin Ströker, Juan Sieira, Pedro Brugada, Mark La Meir, Gian-Battista Chierchia, Carlo De Asmundis, Fabrizio Guarracini

**Affiliations:** 1 Department of Cardiology, S. Chiara Hospital, 38122 Trento, Italy; 2 Heart Rhythm Management Centre, Postgraduate Program in Cardiac Electrophysiology and Pacing, Vrije Universiteit Brussel, Universitair Ziekenhuis Brussel, 1050 Brussels, Belgium; 3 Department of Cardiac Surgery, S. Chiara Hospital, 38122 Trento, Italy; 4 Cardiac Surgery Department, Vrije Universiteit Brussel, Universitair Ziekenhuis Brussel, 1050 Brussels, Belgium

**Keywords:** atrial arrhythmias, atrial fibrillation, hybrid ablation, atrial fibrillation ablation

## Abstract

Electrical isolation of pulmonary veins (PVI) is the cornerstone of invasive treatment of atrial fibrillation (AF). However, arrhythmia-free survival of a PVI only approach is suboptimal in patients with persistent and long-term persistent AF. Hybrid AF ablation has been developed with the aim of combining the advantages of a thoracoscopic surgical ablation (direct visualization of anatomical structures to be spared and the possibility to perform epicardial lesions) and endocardial ablation (possibility to check line block, confirm PVI, and possibility to perform cavotricuspid isthmus ablation). Patient selection is of utmost importance. In persistent and long-term persistent AF, hybrid AF ablation demonstrated promising results in terms of AF free survival. It has been associated with a relatively low complication rate if performed in centers with expertise in hybrid procedures and experience with both surgical and endocardial ablation. Different techniques have been described, with different approaches and lesion sets. The aim of this review is to provide a state-of-the-art overview of hybrid AF ablation.

## 1. Introduction

Electrical isolation of pulmonary veins (PVI) has become the main strategy in the invasive treatment of atrial fibrillation (AF) since the role of atrial extrasystoles originating from the pulmonary veins (PVs) has been demonstrated by Haissaguerre et al. [1]. In particular, endocardial circumferential PVI catheter ablation (CA), with different sources of energy, has gained success because of its safety and efficacy [2,3,4].

However, the likelihood of achieving long-term arrhythmia-free survival differs considerably, depending on the type of underlying AF (paroxysmal vs. persistent AF) and on the presence of risk factors. In patients with persistent and long-term persistent AF, the recurrence rate after the PVI-only procedure is higher and the time between the first AF episode and ablation is a predictor of recurrence [5,6,7,8].

Before the endocardial CA of AF, the first successful invasive treatment for AF was surgical ablation (SA), described by the pioneering work of Dr James Cox in 1987 and further refined as the Cox maze procedure [9]. Although showing good results, SA was limited by the open chest approach. The original Cox maze procedure carried low risk but subsequent studies reported a higher complication rate [10]. The development of thoracoscopic approaches has shed light on new possibilities for AF ablation under direct surgical visualization and without the risk of open chest surgery.

The suboptimal results of CA, especially in the persistent AF subgroup, have led to a quest for new techniques. Hybrid AF ablation has been developed with the aim of combining the advantages of a thoracoscopic SA (direct visualization of anatomical structures to be spared and possibility to deliver epicardial lesions) and endocardial CA (possibility to check line block, confirm PVI and, if necessary, completing lesions from endocardium or performing lesions in areas not accessible by SA).

The aim of this review is to provide a state-of-the-art overview of hybrid AF ablation.

## 2. Rationale of Hybrid Ablation of Atrial Fibrillation

The rationale for the hybrid ablation approach in the treatment of atrial fibrillation lies in its complex physiopathology. Indeed, the suboptimal results of the PVI-only approach in non-paroxysmal AF can be explained by drivers or triggers, or substrates, outside PVs, that may sustain the arrhythmia [11]. These areas outside the PVs can be found in: (1) left atrial posterior wall (LAPW); (2) left atrial appendage (LAA); (3) crista terminalis; (4) interatrial septum (IAS); (5) coronary sinus (CS) and vein of Marshall; (6) superior vena cava (SVC) [12]. Based upon these premises, meta-analyses of randomized trials and observational studies have suggested a benefit of PVI + LAPW isolation (LAPWI) compared to PVI-only in persistent AF [13,14]. However, endocardial LAPWI is limited by the anatomical proximity to the esophagus, and atrio-esophageal fistula, although rare (<0.1%), is the most devastating consequence of esophageal thermal injury during LAPWI [15]. Furthermore, LAPWI can be hampered by epicardial connections, such as the septopulmonary bundle, which can be a difficult target for endocardial CA [16]. Finally, the evidence for endocardial–epicardial dissociation suggests that endocardial-only mapping and ablation may be insufficient to adequately address abnormalities in AF on both cardiac surfaces [17,18]. The role of epicardial substrate in persistent AF extends also to the Marshall vein and targeting this anatomical structure has been demonstrated in the MARSHALL-PLAN study to improve clinical outcomes, if associated with a standard set of radiofrequency lesions (vein of Marshall, coronary sinus, roof, mitral and cavo-tricuspid isthmus) [19].

Hybrid AF ablation, delivering epicardial lesions under direct visualization, can target epicardial substrate (including septopulmonary bundle and Marshall vein) while sparing anatomical structures such as nerves and esophagus. LAA exclusion via epicardial approach may contribute to reducing both arrhythmic recurrences and risk of stroke [9]. Furthermore, hybrid ablation, via endocardial approach, allows ablating the cavo-tricuspid isthmus, a part of the MARSHALL-PLAN lesion set. The endocardial approach is also useful for mapping, checking lines, and touch-up ablation.

## 3. Current Guidelines

Current ESC guidelines on AF [20] recommend thoracoscopic, including hybrid surgical ablation, procedures in patients who have symptomatic, paroxysmal, or persistent AF refractory therapy. In particular, these patients need to have failed percutaneous AF ablation or have evident risk factors for catheter ablation failure in maintaining long-term sinus rhythm (Class IIa, B) [20]. Furthermore, thoracoscopic, including hybrid surgical ablation procedures, may be considered in patients with persistent AF with risk factors for recurrence, who remain symptomatic during AF despite at least one failed antiarrhythmic drug, and who prefer further rhythm control therapy (Class IIb, C) [20].

## 4. Preprocedural Screening

Preprocedural screening for hybrid AF ablation is a crucial step. Patient selection criteria should be carefully discussed by a multidisciplinary team, combining the expertise of cardio-thoracic surgery and electrophysiology [21].

Patients eligible for ablation should be symptomatic, refractory to antiarrhythmic drugs, and, preferentially, with persistent or long-standing persistent atrial fibrillation [21]. Even if not mandatory, most of the patients referred for hybrid AF ablation had previous procedures, including previous PVI. Currently, a hybrid procedure as the first approach is not recommended due to the high success rate of endocardial PVI with low complication rates [21]. However, in the setting of long-standing persistent atrial fibrillation, the CONVERGE trial has shown the superiority of the hybrid convergent approach as a first-line strategy compared with endocardial catheter ablation [22].

Preprocedural screening should rule out absolute and relative contraindications to hybrid AF ablation. Absolute contraindications include the following: left atrial appendage thrombus, previous sternotomy or heart surgery, pregnancy, esophagitis, acute infection, need for concomitant cardiac surgery, and myocardial infection in the last 90 days [21]. Relative contraindications are as follows: left atrial size more than 70 cm^2^, BMI more than 45, connective tissue disorders, advanced liver disease, and history of thoracic radiation therapy [21].

After the initial selection, further preadmission testing may be considered for additional screening. Preprocedural computed tomography (CT) or cardiovascular magnetic resonance (CMR) should be performed to guide catheter ablation. The benefit of CT/CMR includes the ability to evaluate the number, size, and shape of PVs [23]. Evaluation of PV anatomy allows screening of abnormal PVs position that could be an absolute contraindication to hybrid catheter ablation [23]. Unexpected relevant findings on CT/CMR during pre-procedural screening affect clinical management and decision-making. Ebert et al. [24] described extracardiac abnormalities in 1.5% of CT/CMR performed in the context of preprocedural screening for AF catheter ablation. Preprocedural imaging allows us to define prognostic factors; in particular, the left atrium volume, in addition to its importance as an exclusion criterion, is also a reliable risk factor for recurrence [25].

Transthoracic and transesophageal echocardiography allows for evaluating mitral regurgitation, left atrial or left atrial appendage thrombus, left atrial size, and left ventricular ejection fraction.

ICD therapies should be turned off before the procedure. Pacemaker reprogramming should be considered before the procedure on an individual basis.

Anticoagulation should be assessed. Anticoagulation should be maintained for 3–4 weeks before the procedure [21]. An ACT of 300 to 400 s was maintained during the endocardial catheter ablation procedure. Systemic anticoagulation therapy post-procedure should be continued for at least two months. Decisions regarding anticoagulation beyond two months should be based on the patient’s risk factors for stroke and not on the presence or type of AF [26].

A prophylactic regimen of steroids (or nonsteroidal medications) should be considered to prevent pericarditis or Dressler syndrome, or other inflammatory diseases with pericardial effusions if the patient is able to tolerate such a regimen [22]. The use of steroids might interfere with the maturation of the atrial lesions and affect AF recurrence. On the other hand, inflammation plays a role in AF. A meta-analysis suggested that periprocedural administration of corticosteroids after catheter ablation was associated with a reduction in early but not late recurrence of AF [27].

## 5. Procedural Workflow and Techniques

**Figure 1:**
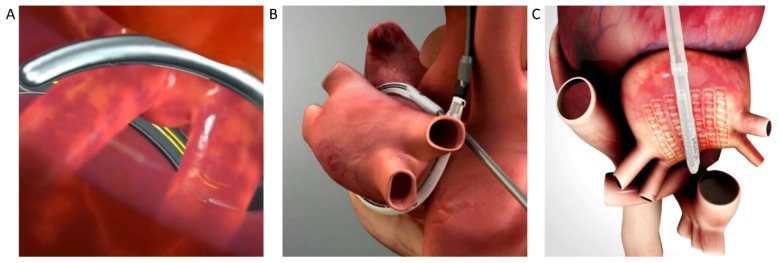
Videothoracoscopic Surgical Techniques overview. Panel (**A**): clamp technique; Panel (**B**): fusion technique; Panel (**C**): convergent technique.

**Figure 2:**
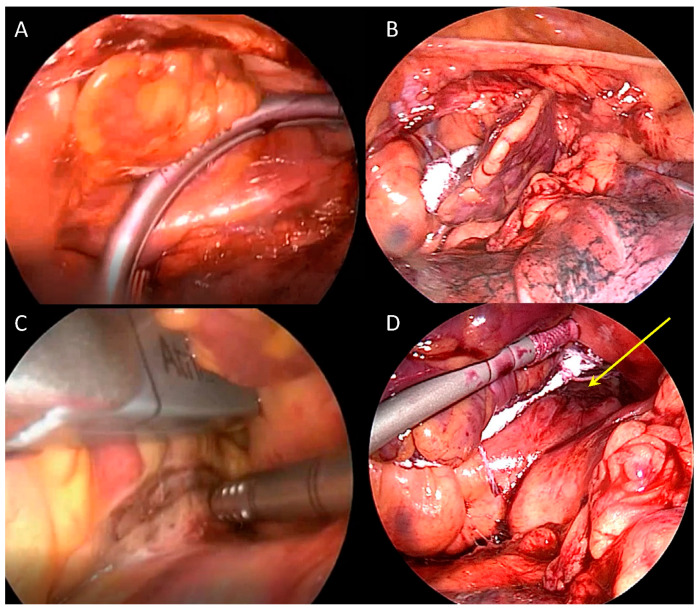
Hybrid AF ablation thoracoscopic access. Panel (**A**): left pulmonary veins isolation with the clamp (Synergy System; Atricure; West Chester, OH, USA); Panel (**B**): left atrial appendage closure with the clip (Atriclip, Atricure, West Chester, OH, USA); Panel (**C**): roof line with linear radiofrequency probe (Coolrail; Atricure, West Chester, OH, USA); Panel (**D**): ablation line between the clip and left pulmonary veins (yellow arrow).

Hybrid AF ablation is based on sequential or combined radiofrequency energy catheter ablation and totally thoracoscopic unilateral/bilateral mono or bipolar radiofrequency ablation on the beating heart. The different techniques are summarized in Figure 1 and Figure 2.

### 5.1. Transvenous Radiofrequency Catheter Ablation

The ablation procedure is performed while the patient is under sedation or general anesthesia. A coronary sinus catheter is placed under fluoroscopy. Access to the left atrium is achieved via a femoral approach and a single or double transseptal puncture. A single bolus of 100 IU/kg bodyweight of heparin is administered, followed by heparin perfusion to maintain an activated clotting time (ACT) above 300 s. An electroanatomic map of the left atrium is created using a multipolar mapping catheter with direct visualization on a three-dimensional mapping system. The ablation is generally performed using an irrigated tip contact force radiofrequency (RF) ablation catheter. Automated lesion tagging can be used to mark the location of each ablation lesion. The minimum lesion set that must be performed is the PVI; other additional lesions (roof line, mitral isthmus line, and posterior line) are added at the electrophysiologist’s discretion. In cases of a documented right atrial flutter (AFL) or AFL induced during the ablation, a cavo-tricuspid isthmus line is performed endocardially. Complete isolation (entrance and exit block) of all PVs and the box lesion, if performed, and completeness of additional ablation lines must be confirmed. 

### 5.2. Surgical Thoracoscopic Epicardial Ablation

Nowadays, thoracoscopic epicardial ablation can be performed in three different ways: (1) bipolar radiofrequency clamp technique; (2) mono and bipolar radiofrequency fusion technique; (3) epicardial ablation with the vacuum-assisted, unipolar radiofrequency technique or convergent technique. All these ablation techniques can be completed with the left atrial appendage occlusion via thoracoscopic approach.

Clamp Technique (Unilateral or Bilateral).

### 5.3. Unilateral Approach

In the unilateral left-sided thoracoscopic approach, under general anesthesia and single lung ventilation, three ports of 5 mm are placed at III, V, and VII intercostal space between anterior and mid axillary lines (for optical fiber and working tools). After opening the pericardial reflections between the inferior vena cava and right inferior pulmonary vein, superior vena cava, and right superior pulmonary vein, and the reflection between oblique and transverse sinus, the left PVs first and then right PVs are encircled with a dedicated tool (Lumitip Atricure; West Chester, OH, USA) and clamped by aid of a tunneled rubber glide path connected to a biparietal bipolar radiofrequency clamp (Synergy System; Atricure, West Chester, OH, USA), Figure 2A. Antral pulmonary veins transmural lesion is achieved when the conduction graph drops within 5 s with a minimum of 6 applications [28]. Linear roof and floor lines in the transverse and oblique sinus, respectively, are performed with the dedicated internally cooled bipolar electrodes device specifically designed to produce continuous full-thickness lesions (Coolrail; Atricure, West Chester, OH, USA), Figure 2C. This technique has the advantage of only 3 ports and single lung ventilation. Disadvantages lie in the unilateral access, which might preclude safe right superior pulmonary vein encircling, targeting of the superior vena cava, or more extensive right-side ablation.

### 5.4. Bilateral Approach

Under general anesthesia starting from the right side and subsequently moving to the left, three ports on each side are placed for camera and working tools, the pericardium is opened on both sides and then partially closed before switching to other side. To access the PVs and all targets of the ablation, direct-view blunt dissection of the pericardial reflections is performed. Ablation may be then performed by a combination of dedicated right and left-shaped bipolar clamps and a rail device (Synergy Clamp and Coolrail; Atricure, Manson, OH, USA) [29] or by a single device bipolar-biparietal internally irrigated radiofrequency overlapping clamps encircling sequentially en-block PVs and the left atrial posterior wall resulting in a complete box lesion (Medtronic Cardioblate Gemini, Medtronic, Minneapolis, MN, USA) with a minimum of 4 applications [30]. Full thickness lesion is guided by a computer impedance-based algorithm for all devices whereas contiguity is assessed by entrance and exit block by a multipolar catheter positioned epicardially through one of the ports in all cases despite the ablating tool used. Disadvantages include a longer duration and potentially higher rate of complications due to a more extensive surgical preparation and bilateral sequential single lung ventilation. However, this approach allows targeting superior vena cava, inferior vena cava, and additional lines approaching the original MAZE IV procedure.

#### 5.4.1. Fusion Technique

The procedure is a right-side closed chest thoracoscopic unilateral epicardial radiofrequency ablation procedure. The transverse and oblique sinuses are opened with blunt dissection. Suction-assisted, mono, and bipolar epicardial ablation is performed after removal of epicardial fat with heparin given to maintain an ACT greater than 300 s. The epicardial fat is removed from the roof of the left atrium and interatrial groove under direct thoracoscopic vision using a mixture of dissection with thoracoscopic scissors and traction on the fat with diathermy until the left atrial muscle is denuded of fat and the left atrium is clearly visible. The aim is to produce a box lesion set encircling en-block all four PVs and posterior left atrial wall by a continuous temperature-controlled internally cooled radiofrequency device with suction adherence (Fusion; Atricure, West Chester, OH, USA), Figure 1B. The alleged advantage of this device is to create a vacuum by sucking the atrial wall into the device.

#### 5.4.2. Convergent Technique

This process is also called the “Hybrid Convergent procedure”, and bases its rationale on the beneficial role of posterior wall isolation in specific subcategories of atrial fibrillation patients [31] and it is the only true obligatory hybrid approach up to date. It requires a subxiphoid stab wound incision and a pericardiotomy to introduce the vacuum assisted unipolar RF probe (Episense; Atricure, West Chester, OH, USA) through a pericardioscopic cannula with an endoscope into the pericardial sac to ablate first the left atrial posterior wall with several contiguous and parallel lesions across the accessible part of the left atrium and then in proximity of left and right PVs antra (Figure 1C). The device integrates a continuous impedance monitoring system to ensure continuous lesions and sensing electrodes to help guide energy delivery and detection of lesion completeness during the epicardial ablation and prior to endocardial ablation. This subxiphoid approach easier for the surgeon, as no thoracoscopic pericardial reflection dissection is required, requires mandatory endocardial transcatheter touch up in order to achieve a comprehensive lesion pattern [26].

### 5.5. Thoracoscopic Left Atrial Appendage Occlusion

**Figure 3:**
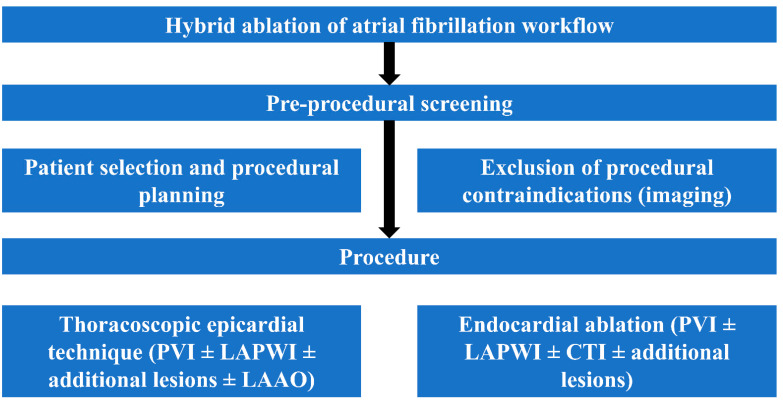
Hybrid AF ablation workflow overview.

Left atrial appendage occlusion (LAAO) has been part of the MAZE procedure since 1991 [32]; its role as a trigger for arrhythmias has been documented by Di Biase et al. in the BELIEF trial [33] and recently confirmed by a metanalysis of Romero J et al. [34] with a 46% relative risk reduction and 22.9% absolute risk reduction of atrial arrhythmia recurrence when electrically isolated. LAA catheter-based electrical isolation is time consuming and technically demanding also in experienced centers and in case of sinus rhythm restoration only with concomitant exclusion of the LAA, anticoagulants may be safely discontinued in the majority of treated patients [35]. There are only two devices specifically designed for epicardial LAAO, the Lariat (Atricure, West Chester, OH, USA) requiring combined endo-epicardial approach and the Atriclip (Atricure, West Chester, OH, USA), the most investigated and implanted, designed for standalone totally thoracoscopic approach; both have been shown to provide effective LAA electrical isolation [36]. Based upon this evidence, all hybrid procedures despite the technique selected are usually completed with LAAO clipping in a 15 min simple procedure performed through three ports of 5 mm in a “hockey stick” figure between anterior and midaxillary line on left hemithorax requiring only basic thoracoscopic skills and providing satisfactory results (Figure 2B). Recently, in the LAAOS III trial, LAAO performed during cardiac surgery undertaken for other reasons demonstrated to reduce stroke or systemic embolism (hazard ratio, 0.67; 95% confidence interval, 0.53 to 0.85; *p* = 0.001) [37]. Complete workflow of hybrid AF ablation is summarized in Figure 3.

## 6. Clinical Outcomes and Future Perspectives

Over the past decade, there has been great interest in hybrid AF ablation, based on the promising clinical results. Indeed, the idea to achieve a minimally invasive surgical approach replicating the Cox maze procedure is appealing [32]. Although the literature is sparse, there is considerable evidence supporting the feasibility and safety of this procedure. An overview of clinical studies is provided in Table 1.

In 2005, Wolf et al. [38] reported the first results of 21 patients treated with a minimally invasive surgical approach to PVI. In their work, the authors described the feasibility of video-assisted thoracoscopic PVI with excision of the left atrial appendage in a new promising minimal approach.

Since the first publications, the experience with minimally invasive surgery has increased with the development of hybrid procedures for the treatment of AF.

In this regard, Mahapatra et al. [39] described their experience with a staged hybrid AF ablation on 15 patients with persistent and long-standing persistent AF who had failed catheter ablation.

They reported how a sequential minimally invasive epicardial surgical ablation, through a bilateral thoracoscopic approach, followed by endocardial catheter-based ablation, had a higher early success rate than repeat catheter ablation alone. In particular, freedom from atrial arrhythmias in the hybrid group vs. matched catheter ablation group at 20 months of follow-up was 87% vs. 53%, respectively.

La Meir et al. [40] compared a hybrid vs. a standard surgical bilateral thoracoscopic approach employing radiofrequency (RF) sources in the surgical treatment of AF. In the hybrid group, a mitral isthmus line and superior vena cava isolation were added endocardially in addition to PVI, posterior wall isolation, and ganglionic plexi ablation during the same procedure. At 1-year follow-up, the hybrid group produced better results (8.2% vs. 14.9%, *p* = 0.04).

The same research group also reported how in 64 patients undergoing hybrid AF ablation, combining a transvenous endocardial approach and a thoracoscopic epicardial approach in a single procedure, resulted in 3-year cumulative freedom from arrhythmia without antiarrhythmic drugs or redo ablation of 80% in paroxysmal AF and 79% in non-paroxysmal AF [41].

In 2012, Pison et al. [42] reported data after 1 year following a hybrid procedure in 26 patients. The hybrid approach was performed in patients with previously failed catheter ablation, left atrial volume ≥29 mL/m^2,^ and persistent or longstanding persistent AF. After PVI through a bilateral thoracoscopic approach, an endocardial mapping to confirm PVI was created in all patients. Additional posterior box isolation, superior vena cava isolation, intercaval line, and mitral isthmus line were performed in patients with non-paroxysmal AF. At 1-year follow-up, the single-procedure success rate was 83% (93% in paroxysmal AF versus 90% in non-paroxysmal AF). No deaths or conversion to cardiopulmonary bypass were reported. One patient had a pleural effusion, and one patient was hospitalized for chest pain at the insertion sites of the working ports. In a second case series, Pison and collaborators reported the outcome of the same hybrid approach in 78 patients after 2 years of follow-up [43]. Freedom from atrial arrhythmias off antiarrhythmic drugs (AADs) was 87% and 92% on AADs. In this study, the complication rate was 8%.

To our knowledge, the HARTCAP-AF trial is the only ongoing randomized trial that compares hybrid AF ablation (thoracoscopic surgical clamp technique + transvenous catheter ablation) versus transvenous catheter ablation in the treatment of persistent AF [44]^.^

Another described approach involves an endocardial evaluation not concomitant but sequential to the minimally invasive surgical procedure.

Bulava et al. [45] in their experience with a staged hybrid AF ablation, described freedom from atrial arrhythmia off AADs of 94% and 84% on AADs at 1-year follow-up. The staged procedure consisted of a surgical radiofrequency thoracoscopic ablation (PVI, posterior box, trigone line, ganglionic plexi ablation, and LAA exclusion) followed by radiofrequency catheter ablation after 6–8 weeks to verify/complete previous epicardial ablation. 

Muneretto et al. [46] (HISTORIC-AF trial, multicenter, prospective, single-arm study) described the feasibility of their sequential tailored hybrid approach in persistent AF patients. The surgical procedure consisted of an epicardial ablation performed exclusively via a right monolateral thoracoscopic approach (Fusion Technique), whereas the transcatheter procedure was performed at the end of the 3 months blanking period. At 12-month follow-up, a stable sinus rhythm was achieved in 88% of patients treated with hybrid ablation.

More recently, Richardson et al. [47] compared the effects of the timing of the hybrid procedure on AF recurrence. In the study, the authors retrospectively analyzed 83 patients undergoing staged hybrid AF ablation versus simultaneous ablation. Although staged hybrid ablation significantly increased the likelihood of incomplete PVI diagnosis at the time of endocardial mapping, the stepwise approach did not improve the time to first AF recurrence.

In 2020, the first multicenter randomized controlled trial comparing combined epicardial and endocardial ablation vs. endocardial catheter ablation only for the treatment of persistent AF patients was published [26]. In the trial, De Lurgio et al. showed the superiority of a minimally invasive epicardial/endocardial ablation approach compared with standard endocardial catheter ablation for the treatment of persistent and long-standing persistent atrial fibrillation. One-hundred and fifty-three patients were randomized 2:1 to hybrid convergent procedure versus endocardial catheter ablation. In the catheter ablation group, PVI, roofline, and cavo-tricuspid isthmus ablation were performed. Moreover, complex fractionated atrial electrogram ablation was left to physician discretion if the patient did not convert after the other mandatory lesions were created. At 1-year follow-up freedom from atrial tachyarrhythmias on AADs was achieved in 67.7% (67/99) patients with hybrid convergent vs. 50.0% (25/50) with catheter ablation (*p* = 0.036) and in 53.5% (53/99) vs. 32.0% (16/50; *p* = 0.0128) off AADs. Moreover, at 18 months using 7-day Holter, 74% of patients in the hybrid convergent arm achieved at least 90% AF burden reduction when compared to 55% with endocardial catheter ablation only (risk ratio, 1.34, *p* = 0.0395).

The complication rate of hybrid AF ablation is about 6.5% [48]. Complications include the following: mortality (0.2%), stroke (0.3%), bleeding (1.6%), conversion to sternotomy (0.3%), phrenic nerve injury (0.3%), pacemaker implantation (0.6%), and atrio-esophageal fistula (0.4%), Table 2.

Pulsed field ablation (PFA) is emerging as a nonthermal ablative modality in which ultrarapid electrical fields are applied to target tissue. The threshold field strength that induces necrosis is different for various tissues, thus improving selectivity and safety [49]. To date, there are no studies on PFA in the context of hybrid AF ablation. However, with the new technological development in the hybrid thoracoscopic field, the idea of integrating the advantages of PFA energy into a novel tool appears appealing.

## 7. Conclusions

Despite the technological advancements in the field of AF ablation, long-term arrhythmia-free survival heavily depends on the type of underlying AF, and patients with persistent and long-term persistent AF have a bad prognosis. Hybrid AF ablation combines both thoracoscopic SA and endocardial CA advantages. It shows promising results in terms of AF-free survival with a relatively low complication rate if performed in centers with expertise in hybrid procedures and experience with both SA and CA.

## Figures and Tables

**Table 1:** Summary of studies on hybrid atrial fibrillation ablation.

Reference	Study Category	N. of Patients	Paroxysmal AF/Persistent AF (n)	First Procedure (+/−)	Surgical Technique	Lesions Performed	Complications	Follow-Up (Months)	Freedom from AF
Wolf et al., 2005 [38]	Observational	27	18/9	+	Bilateral	PVI + LAAc	3 minor, 3 major	6	91.3%
Mahapatra et al., 2011 [39]	Observational	15	0/15	−	Unilateral	PVI, roof line, mitral line, LAAc, GPa	0	20.7	86.7%
La Meir et al., 2013 [40]	Observational	63	35/0	+	Unilateral	PVI, inferior line, roof line, isthmus, endocardial, LAAc	0	12	91.4%
Maesen et al., 2018 [41]	Observational	64	30/34	+(66%)	Unilateral	PVI, roof line, inferior line, LAAc	2 minor, 1 major	36	80%
Pison et al., 2012 [42]	Observational	26	15/11	+	Unilateral	PVI, CTI, SVCi, intercaval line, mitral line	1 minor	12	83%
Pison et al., 2014 [43]	Observational	78	29/49	+(68%)	Unilateral	PVI, roof line, inferior line, mitral line, CTI, intercaval line, LAAc, GPa	6 minor	12	82% (persistent AF), 76% (paroxysmal AF)
Bulava et al., 2015 [45]	Observational	50	0/50	+	Bilateral	PVI, roof line, inferior line, LAAc, intercaval line, GPa	7 major, 10 minor	12	94%
Richardson et al., 2016 [47]	Observational	83	0/83	+	Bilateral	PVI, roof line, inferior line, intercaval line, LAAc	6 minor, 1 major	12	71%
Muneretto et al., 2017 [46]	Observational	100	0/100	+(45%)	Fusion	Box Lesion	3 minor, 3 major	12	88%
De Lurgio et al., 2020 [26]	RCT	102	0/102	+	Subxiphoid	PVI, PWI, CTI	8 major	12	67.7%

AF, atrial fibrillation; PVI, pulmonary vein isolation; LAAc, left atrial appendage closure; GPa, ganglionic plexi ablation; SVCi, superior vena cava isolation; RCT, randomized controlled trial.

**Table 2:** Summary of complications and management strategies.

Complication	Complication Rate	Management
Mortality	0.2%	-
Stroke	0.3%	Conservative/interventional
Severe bleeding	1.6%	Reintervention
Cardiac perforation	0.3%	Conversion to sternotomy
Phrenic nerve injury	0.3%	Conservative (can be transient)
Atrio-ventricular block or sinus node dysfunction	0.6%	Permanent pacemaker implantation
Atrio-esophageal fistula	0.4%	Surgery

## Data Availability

Not applicable.
